# Design Variation of a Dual-Antigen Liposomal Vaccine Carrier System

**DOI:** 10.3390/ma12172809

**Published:** 2019-09-01

**Authors:** Roozbeh Nayerhoda, Andrew Hill, Marie Beitelshees, Charles Jones, Blaine Pfeifer

**Affiliations:** 1 Department of Biomedical Engineering, University at Buffalo, The State University of New York, Buffalo, NY 14260, USA; 2 Department of Chemical and Biological Engineering, University at Buffalo, The State University of New York, Buffalo, NY 14260, USA; 3 Abcombi Biosciences Inc., 1576 Sweet Home Road, Amherst, NY 14228, USA

**Keywords:** liposome, encapsulation, delivery, vaccine, pneumococcal disease

## Abstract

The enclosed work focuses on the construction variables associated with a dual-antigen liposomal carrier, delivering encapsulated polysaccharides and surface-localized proteins, which served as a vaccine delivery device effective against pneumococcal disease. Here, the goal was to better characterize and compare the carrier across a range of formulation steps and assessment metrics. Specifically, the vaccine carrier was subjected to new methods of liposomal formation, including alterations to the base components used for subsequent macromolecule encapsulation and surface attachment, with characterization spanning polysaccharide encapsulation, liposomal size and charge, and surface protein localization. Results demonstrate variations across the liposomal constructs comprised two means of surface-localizing proteins (either via metal or biological affinity). In general, final liposomal constructs demonstrated a size and zeta potential range of approximately 50 to 600 nm and −4 to −41 mV, respectively, while demonstrating at least 60% polysaccharide encapsulation efficiency and 60% protein surface localization for top-performing liposomal carrier constructs. The results, thus, indicate that multiple formulations could serve in support of vaccination studies, and that the selection of a suitable final delivery system would be dictated by preferences or requirements linked to target antigens and/or regulatory demands.

## 1. Introduction

Vaccines have a significant impact on overall global health and are expected to continue to serve as key prophylactic options against current and emerging infectious diseases and cancer [1,2,3,4]. Traditionally, vaccine formulations stressed antigen provision as the key ingredient required for initiating an immune response cascade (often accompanied by an adjuvant or similar supporting ingredient to prompt immunogenicity) [5,6]. For many types of diseases, the correct antigen, linked to the key aspect of a disease, is sufficient to prompt a protective immune response [7].

However, for certain diseases, vaccine design and delivery can play a deciding role in ultimate effectiveness [8]. An excellent example is the case of pneumococcal disease [9]. The bacterium *Streptococcus pneumonia* (*S. pneumonia*) is responsible for pneumococcal disease, which initiates upon *S. pneumoniae* establishing an asymptomatic biofilm in a human host [10,11]. The original antigen used as the basis for a vaccine against pneumococcal disease was obtained from capsular polysaccharides of *S. pneumoniae* that are prominent during the colonization stage of infection [12]. The polysaccharides alone, however, are suboptimal in triggering a long-lasting and fully protective immune response [13,14]. As such, a more potent vaccine was designed in which the polysaccharide antigen was conjugated covalently to an immunogenic protein [15,16]. The resulting pneumococcal conjugate vaccines, via the inclusion of a conjugated protein carrier, trigger an enhanced and longer-lasting antibody response to subsequent disease challenge [17].

**Figure 1:**
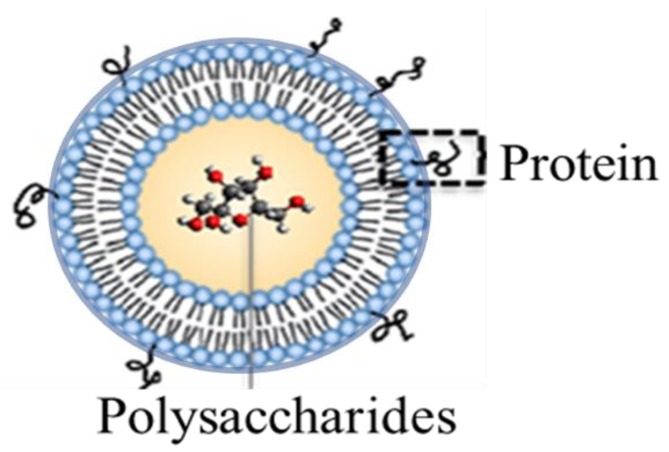
Dual-antigen delivery liposomal vaccine carrier featuring encapsulated polysaccharides and surface-attached proteins.

In response to challenges posed by the chemical conjugation techniques associated with current pneumococcal vaccines (which pose economic limits in the chemical conjugation steps required for broad immunization), we developed a liposomal vaccine carrier that serves multiple purposes [18,19]. Firstly, as illustrated in Figure 1, the carrier has the ability to both encapsulate polysaccharide content and non-covalently localize protein units to the liposomal surface. By doing so, the liposome mimics the conjugation effect associated with current pneumococcal vaccines in provoking a long-lasting and potent immune response. The dual-antigen format also allows for a simpler and more expansive means of including numerous polysaccharide and protein components important in both extending coverage breadth of the vaccine (through the incorporation of polysaccharide antigen variants from unique *S. pneumoniae* strains (termed serotypes) and accounting for disease progression associated with pneumococcal disease (via the simple non-covalent surface localization of protein antigens capable of directing immune reactivity to multiple stages of bacterial pathogenesis). Finally, the noncovalent protein attachment mechanisms associated with the liposomal construct, based upon either metal chelation or biotin affinity, contribute to the flexibility of polysaccharide/protein inclusion and interchange, and they economically simplify the final vaccine formulation relative to current approaches that require covalent chemical conjugation.

In this work, the dual-antigen liposomal vaccine carrier was tested across a range of formulation parameters to better characterize and improve functionality. In particular, upon being subjected to new formulation steps, several different liposomes possessing distinct surface protein attachment mechanisms were characterized for polysaccharide encapsulation, size, and zeta potential. The products and comparisons, thus, established an updated protocol for liposomal formulation and characterization to be applied to future vaccine delivery efforts.

## 2. Materials and Methods

### 2.1. Materials and Reagents

All liposomal construction materials were obtained from Avanti Polar Lipids, ThermoFisher Scientific, or Sigma Aldrich. Pneumococcal capsular polysaccharide (serotype 19F) was obtained from the American Type Culture Collection (ATCC), and molecular details of the polysaccharide were reported previously [20,21]. Green fluorescent protein (GFP) was produced recombinantly as previously reported [19]; briefly, the GFP protein was generated through gene expression within *Escherichia coli* with the protein product containing a 6× histidine tag to facilitate affinity chromatography using an Ni–nitrilotriacetic acid (NTA) packed-bed column matrix.

### 2.2. Liposomal Preparation

Relative lipid molar amounts for liposomal formulations are presented in Table 1. To prepare the 1,2-dioleoyl-sn-glycero-3-[(*N*-(5-amino-1-carboxypentyl)iminodiacetic acid)succinyl] (DGS)–NTA liposomal component containing the cobalt and zinc metals, lipid was firstly dissolved in chloroform. Either cobalt (II) chloride or zinc (II) chloride solutions (20 mg/mL (in deionized (DI) water)) were added, followed by brief sonication (to mix the solutions) and then dialyzed in 1× phosphate-buffered saline (PBS). The resulting DGS–NTA(Co) or DGS–NTA(Zn) lipid components were then used in the final liposomal formulation process. To the lipid mixtures indicated in Table 1, 1 mL of 0.6 mg/mL polysaccharide 19F was added and vortexed for 1 min, then evaporated using a rotatory evaporator to form a thin film, followed by 1× PBS rehydration at 45 °C using a rotatory evaporator rotated until the thin film was again fully dissolved (Figure S1, Supplementary Materials). The sample was then passed 10 to 12 times through a handheld extruder with a 200-nm pore size membrane.

In order to separate liposomes encapsulating polysaccharide 19F from free polysaccharides, 500 µL of the post-extrusion sample was transferred to a 300-kDa centrifugal tube (Pall Co., Port Washington, NY, USA) and centrifuged for 5 min at 4 °C and 1200 relative centrifugal force (rcf). The resulting filtered sample volume was adjusted to the initial volume using PBS and then subjected to protein binding by incubation with GFP (280 µg) for 30 min at room temperature. To separate unbound protein from GFP-bound liposomes encapsulating polysaccharide 19F, samples were subjected to centrifugation purification, and resulting liposomes were further purified with an additional centrifugation step (Figure S2, Supplementary Materials).

### 2.3. Polysaccharide Encapsulation Analysis

Liposome samples (0.6 mL) were mixed with 3 mL of 5% (*w*/*v*) phenol and 1.5 mL of concentrated sulfuric acid and vortexed for 5 s. The resulting solution (250 µL) was transferred to a Falcon^®^ 96-well microplate, covered, and incubated for 15 min in a 92 °C water bath, followed by incubation at room temperature for the same duration, allowing the well plates to cool to the point of colorimetric analysis at 480 nm using a Synergy Multi-Mode Microplate Reader (BioTek Instruments Inc., Winooski, Vermont, VT, USA). Encapsulated polysaccharide values were calculated by comparison to a standard calibration curve and then ratioed to the initial amount of polysaccharide introduced to the liposomal production process to calculate percentage encapsulation values.

### 2.4. Size and Zeta Potential Analysis

After diluting samples in PBS, dynamic light scattering measurements were made using a Zetasizer Nano ZS90 instrument (Malvern) to determine particle diameter and zeta potential of liposomes at 25 °C with a 4-mW, 633-nm He–Ne laser as the light source at a fixed measuring angle of 90° to the incident laser beam.

### 2.5. Liposomal Protein Surface Assessment

Liposomal samples were assessed for GFP surface binding efficiency via fluorescence analysis at an excitation wavelength of 359 nm and an emission wavelength of 508 nm using a Synergy 4 Multi-Mode Microplate Reader (BioTek Instruments Inc.). Resulting values were compared to a standard GFP calibration curve and ratioed to the initial amount of protein introduced to the liposomal surface binding step to calculate percentage encapsulation values.

### 2.6. Experimental Repetition

Error bars associated with data represent results from three different experimental efforts.

## 3. Results

### 3.1. Liposomal Formulation without Polysaccharide Encapsulation and without Protein Surface Binding

A refined process for liposomal formulation based upon a concept called liposomal encapsulation of polysaccharide (LEPS) [19] was completed in this study with the inclusion (via various lipid components) of several different protein surface binding elements. Specifically, both metal chelation affinity (designed for proteins containing 6× histidine tags) and a biotin moiety (to facilitate streptavidin-based binding) were included [18]. The use of two different protein attachment mechanisms allows for alternative means of protein surface localization in case one offers potential advantages (for example, via improved attachment or improved immune response) in various applications of antigen delivery anticipated for future vaccine studies.

**Figure 2:**
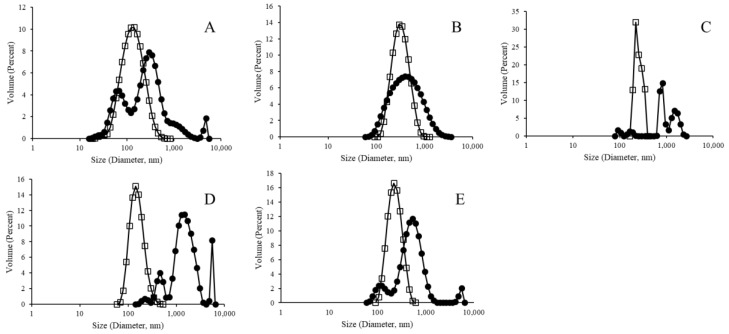
Size distribution analysis of empty liposomes, with closed circles representing pre-extrusion and open squares representing post-extrusion samples for liposomes without a metallic binding element ((**A**) polydispersity index (PDI): 0.62 and 0.31), and with Ni ((**B**) PDI: 0.32 and 0.25), Co ((**C**) PDI: 0.42 and 0.74), Zn ((**D**) PDI: 0.40 and 0.46), and biotinylation ((**E**) PDI: 0.49 and 0.33).

In this study, we sought to better characterize and compare the variety of LEPS particles across parameters of liposomal integrity, polysaccharide encapsulation, and protein surface binding. In particular, we analyzed each step of the liposomal formulation process and evaluated new steps in particle purification in an attempt to refine and better characterize the process of LEPS generation. Initially, however, empty liposomal products were tested for size and zeta potential, as presented in Figure 2 for pre- and post-extrusion formulation samples, that is, early lamellar structures resulting from vesicle formation and final liposomal products resulting from extrusion, respectively (Figure S1, Supplementary Materials). In particular, the resulting data demonstrate the normalizing effect that the extrusion process had upon final formulation size distributions.

Table 2 presents the zeta potential readings for these same sets of liposomal products. Taking the sample without a metallic binding element as a baseline, a stable zeta potential of approximately −33 mV resulted across pre- and post-extrusion samples. Relative to this standard, comparable values (either for the pre- or post-extrusion samples) were observed for the Ni and biotinylated samples, whereas zeta potential values for the Co and Zn samples were noticeably more positive relative to the baseline samples without metallic binding elements.

### 3.2. Liposomal Purification Effect on Liposomal Formulation with Polysaccharide Encapsulation But without Protein Surface Binding

**Figure 3:**
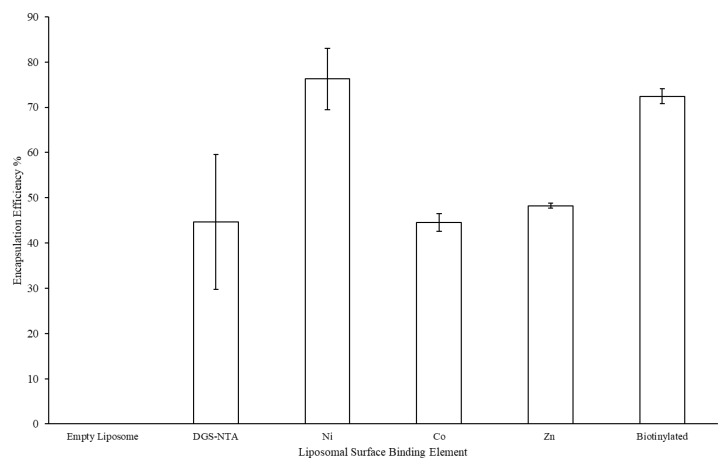
Liposomal encapsulation efficiency of polysaccharide 19F across variants with different protein surface binding elements.

The LEPS formulation process was next tested for the encapsulation of polysaccharide 19F as a representative *S. pneumoniae* serotype capsular polysaccharide. Resulting encapsulation results are presented in Figure 3, with a minimum of 40% polysaccharide encapsulation resulting for all liposomal variants (with analysis based upon an assay optimized for the 19F polysaccharide; Figure S3, Supplementary Materials).

**Figure 4:**
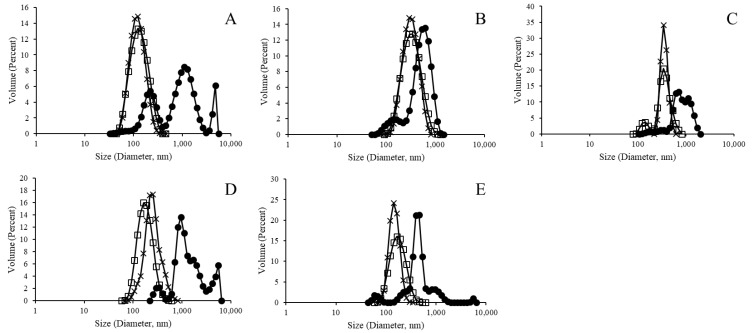
Post-purification size distribution analysis of polysaccharide 19F encapsulated liposomes. Closed circles, open squares, and × signs, respectively, represent pre-extrusion, post-extrusion, and post-purification liposomes without metallic binding element ((**A**) PDI: 0.90, 0.15, 0.29), and with Ni ((**B**) PDI: 0.52, 0.16, 0.24), Co ((**C**) PDI: 0.80, 0.72, 0.43), Zn ((**D**) PDI: 0.59, 0.23, 0.66), and biotinylation ((**E**) PDI: 0.82, 0.175, 0.36).

Prior to efforts of protein surface binding, a purification centrifugation method was applied to the extruded liposomal products (Figures S2 and S4, Supplementary Materials). In so doing, excess unencapsulated polysaccharide remaining from the extrusion formation process (which also provided a degree of liposomal purification) would be removed and a purer liposomal product would be available for the protein surface binding reaction. As Figure 4 indicates, purified liposomal products retained the normalized size distribution pattern observed for the post-extruded liposomal products. Table 3 presents the zeta potential measurements for the purified liposomal products.

### 3.3. Complete LEPS Formulation Characterization after Protein Surface Binding

**Figure 5:**
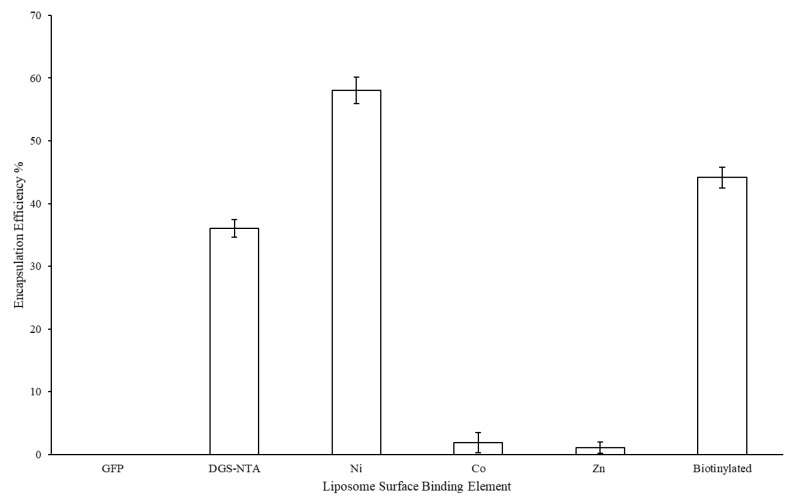
Liposomal encapsulation efficiency of polysaccharide 19F across variants with different protein surface binding elements after protein incubation.

The final series of characterization experiments assessed key metrics associated with macromolecule co-localization, namely, polysaccharide encapsulation and protein surface attachment. Figure 5 indicates the encapsulation efficiency of polysaccharide 19F after the GFP protein was incubated with the purified liposomal products characterized in Section 3.2 (with GFP tested to confirm no background interference with the polysaccharide encapsulation assay). Here, while the samples without any chelating metal, nickel, and biotin showed retained polysaccharide encapsulation, the cobalt and zinc metal chelation samples showed marked reduced polysaccharide content.

**Figure 6:**
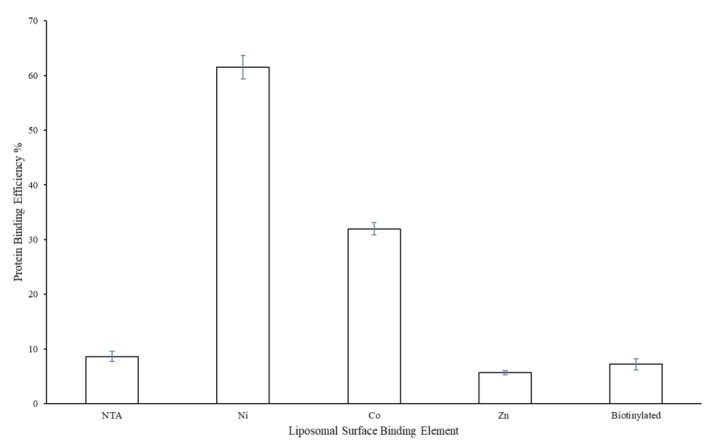
Liposomal surface protein binding efficiency comparison across attachment mechanism variants.

Protein binding to the surface of the liposomal variants is presented in Figure 6. Here, the nickel sample showed the best value for protein binding efficiency. In this analysis, a 6× histidine-tagged GFP protein was used as a model surface attachment protein to facilitate ready fluorescent analysis. As such, surface attachment was only expected for the metal chelation liposomal constructs. Thus, minimal surface attachment was observed for the NTA and biotinylated liposome variants. The cobalt metal liposomal variant showed the second-best protein binding efficiency; however, the zinc liposomal variant showed only minimal surface protein attachment.

**Figure 7:**
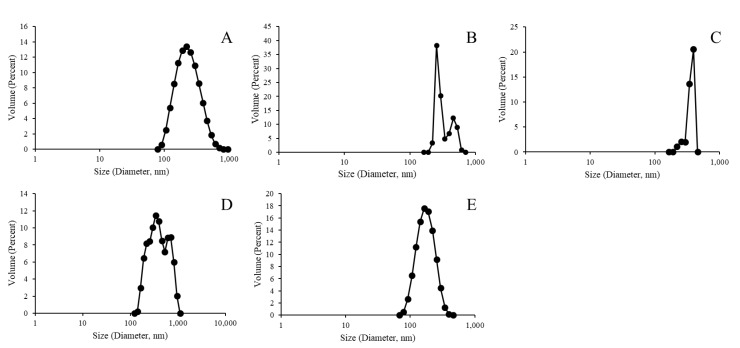
Size distribution analysis of polysaccharide 19F encapsulated green fluorescent protein (GFP)-bound liposomes without metallic binding element ((**A**) PDI: 0.17), and with Ni ((**B**) PDI: 0.25), Co ((**C**) PDI: 0.74), Zn ((**D**) PDI: 0.79), and biotinylation ((**E**) PDI: 0.34).

Complete liposomal products resulting from the protein surface attachment process were finally assessed for particle size distribution as indicated in Figure 7. All samples indicated a size distribution similar to the results presented in Figure 2 and Figure 4; however, more normal distributions were seen for samples without a metal chelation element (i.e., the NTA and biotinylation variants).

## 4. Discussion

The enclosed study provided a comparative analysis across the LEPS delivery system for co-localized polysaccharide and protein antigens. In particular, liposomal formulations were generated for different means of protein surface attachment. In the past, we used both metal chelation and biotinylation to secure protein molecules to the surface of liposomal products [18]. As such, these same samples were now analytically subjected to a stepwise rendition of the LEPS formulation process to compare across standard characteristics that included liposomal size and zeta potential.

Figure 2, Figure 4 and Figure 7 indicate a trend for uniformly normalized size distributions for liposomes formed without metal compounds or with biotin. These results coincide with samples that do not have the ability to bind a His-tagged surface protein (GFP, in this case); thus, the lack of surface perturbation may lead to the more consistent readings for these samples across particle construction. In Figure 2 and Figure 4, the size distribution curves for the nickel and cobalt samples were also uniformly distributed, whereas the curves for zinc samples showed less uniformity.

This result suggests that the zinc liposomes are generally less stable than the other variants tested in this study. Comparisons of zeta potential values across Table 2 and Table 3 similarly support higher variation in values relative to the liposomal samples that do not have binding capacity (i.e., the NTA and biotinylation entries in Table 1). Together, these results and comparisons indicate an intrinsic instability associated with the zinc samples.

Furthermore, the loss of polysaccharide encapsulation for the Co and Zn samples when comparing Figure 3 and Figure 5 (i.e., before and after surface binding attempts) suggests that these samples, in particular, lost structural integrity when subjected to surface membrane perturbation. In the case of the Co sample, evidence of surface binding (Figure 6) suggests that protein attachment caused membrane leakage and the loss of encapsulated polysaccharide. However, in the case of the Zn sample, little protein binding was observed, further suggesting that this sample has less structural integrity overall, which was further diminished upon subjecting to the surface protein binding process. The loss of polysaccharide from the Co sample also highlights the higher variation in zeta potential values (relative to the evenly distributed and/or more stable (via polysaccharide retention) NTA, Ni, and biotinylated samples).

Finally, we note that, while the Ni sample lost the clean uniform size distribution in Figure 7 (relative to the distributions presented in Figure 2 and Figure 3), polysaccharide content was retained (Figure 5). More generally, the differences in size distribution patterns in Figure 7 indicate a lack of liposomal integrity (for the Co and Zn samples), distribution size variation due to protein binding (for the Ni sample), or possibly both (again in the cases of the Co and Zn samples). We also note that, like the NTA sample, the biotinylation sample served as a control without the ability to bind the His-tagged GFP protein. However, the characterization conducted here can similarly be carried out in future efforts to assess protein surface attachment via biotin-streptavidin affinity.

## 5. Conclusions

A liposomal dual-antigen delivery vehicle was extensively characterized across base construction components involved with surface localization of protein agents. Those components studied in this work that were not designed for protein attachment (and, thus, served as controls) demonstrated consistent and uniform polysaccharide encapsulation (≥30% efficiency), size (~100 nm), and zeta potential (approximately −30 mV, although there were variations). Of those liposomes containing metals capable of chelating histidine-tagged proteins (GFP in this study), the nickel sample demonstrated the most consistency across the metrics of size (~200 nm), zeta potential (approximately −30 mV), polysaccharide encapsulation (≥60% efficiency), and protein surface binding (~60% efficiency) and, thus, shows the best potential for utilization as a co-antigen delivery carrier when compared to samples utilizing cobalt or zinc, which resulted in less uniform characterization metrics (generally less uniformly distributed by size with more positive zeta potential values) and decreased polysaccharide retention following protein exposure (≤30% efficiency).

## Figures and Tables

**Table 1:** Liposome composition with regards to binding element.

Binding Element	Lipids or Liposomal Component						
	DOPG	DOPC	DGS–NTA(Ni)	DGS–NTA	DSPE–PEG(2000)	CH	DSPE–PEG(2000)–Biotin
NTA–Ni	3	3	1	0	0.1	4	0
NTA–Co	3	3	0	1	0.1	4	0
NTA–Zn	3	3	0	1	0.1	4	0
NTA	3	3	0	1	0.1	4	0
Biotinylation	3	3	0	0	0	4	0.1

DOPG: 1:2-dioleoyl-*sn*-glycero-3-phospho-(1′-rac-glycerol); DOPC: 1,2-dioleoyl-*sn*-glycero-3-phosphocholine; DGS–NTA(Ni): 1,2-dioleoyl-*sn*-glycero-3-[(*N*-(5-amino-1-carboxypentyl)iminodiacetic acid)succinyl] (nickel salt); DSPE–PEG(2000): 1,2-distearoyl-*sn*-glycero-3-phosphoethanolamine-*N*-[amino(polyethylene glycol)-2000] (ammonium salt); CH: cholesterol; DSPE–PEG(2000)–biotin: 1,2-distearoyl-*sn*-glycero-3-phosphoethanolamine-*N*-[biotinyl(polyethylene glycol)-2000] (ammonium salt).

**Table 2:** Empty liposome surface charge.

Particle	Zeta Potential (mV)
Without metallic binding element pre extrusion	−34.23
Without metallic binding element post extrusion	−33.73
Ni pre extrusion	−46.2
Ni post extrusion	−18.43
Co pre extrusion	−3.42
Co post extrusion	0.01
Zn pre extrusion	−4.52
Zn post extrusion	−7.16
Biotinylated pre extrusion	−36.93
Biotinylated post extrusion	−10.52

**Table 3:** Polysaccharide 19F encapsulated liposome surface charge.

Particle	Zeta Potential (mV)
Without metallic binding element post extrusion	−3.50
Without metallic binding element post purification	−41.16
Ni post extrusion	−41.7
Ni post purification	−29.6
Co post extrusion	−6.04
Co post purification	−22.93
Zn post extrusion	−4.19
Zn post purification	−3.27
Biotinylated post extrusion	−29.7
Biotinylated post purification	−35.06

## Supplementary Materials

The following are available online at https://www.mdpi.com/1996-1944/12/17/2809/s1: Figure S1: Liposomal formation schematic; Figure S2: Liposomal purification and protein surface labeling; Figure S3: Assay for polysaccharide quantification; Figure S4: Purification process for the NTA–cobalt liposomal variant.
