# Supplementary material for: Design Variation of a Dual-Antigen Liposomal Vaccine Carrier System
Source: Materials (Basel). 2019 Sep 1;12(17):2809. doi: 10.3390/ma12172809 (PMC6747791; doi:10.3390/ma12172809)
Supplement: Supplementary file 1 [file materials-12-02809-s001.pdf]

materials

MDPI

Article

Design Variation of a Dual-Antigen Liposomal Vaccine Carrier System

Roozbeh Nayerhoda [1], Andrew Hill [2,3], Marie Beitelshees [2], Charles Jones [3,*] and Blaine Pfeifer [1,2,*]

[1] Department of Biomedical Engineering, University at Buffalo, The State University of New York, Buffalo, NY, 14260, USA
[2] Department of Chemical and Biological Engineering, University at Buffalo, The State University of New York, Buffalo, NY, 14260, USA
[3] Abcombi Biosciences Inc., 1576 Sweet Home Road, Amherst, NY 14228, USA
* Correspondence: charles.jones@abcombibio.com (C.J.); blainepf@buffalo.edu (B.P.)

Supplementary Materials:

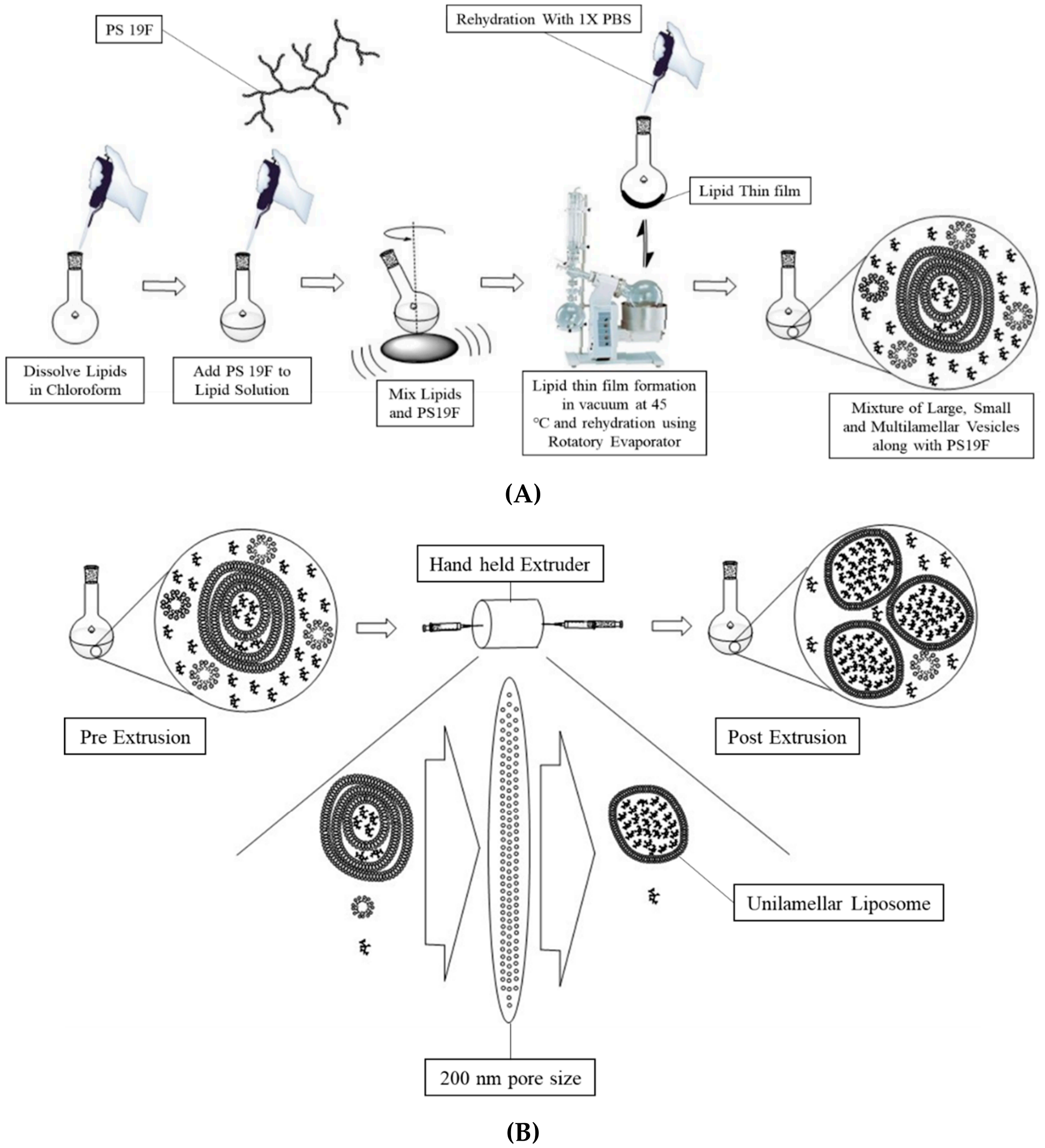

Figure S1. Liposomal formation schematic featuring initiation (**A**) and extrusion (**B**).

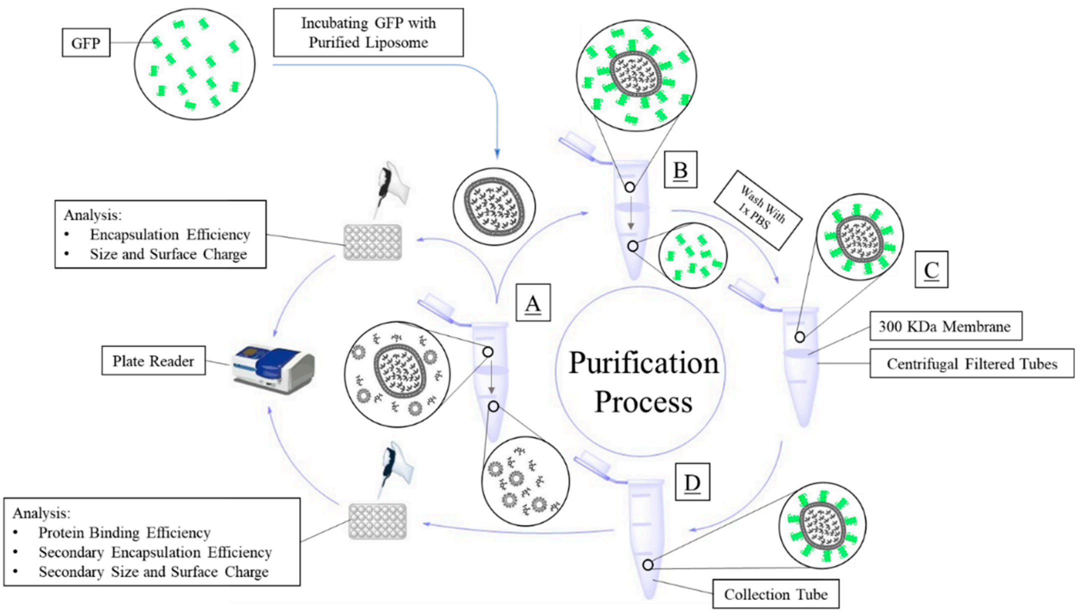

Figure S2. Liposomal purification and protein surface labeling (with GFP, in this case), proceeding from steps A to D.

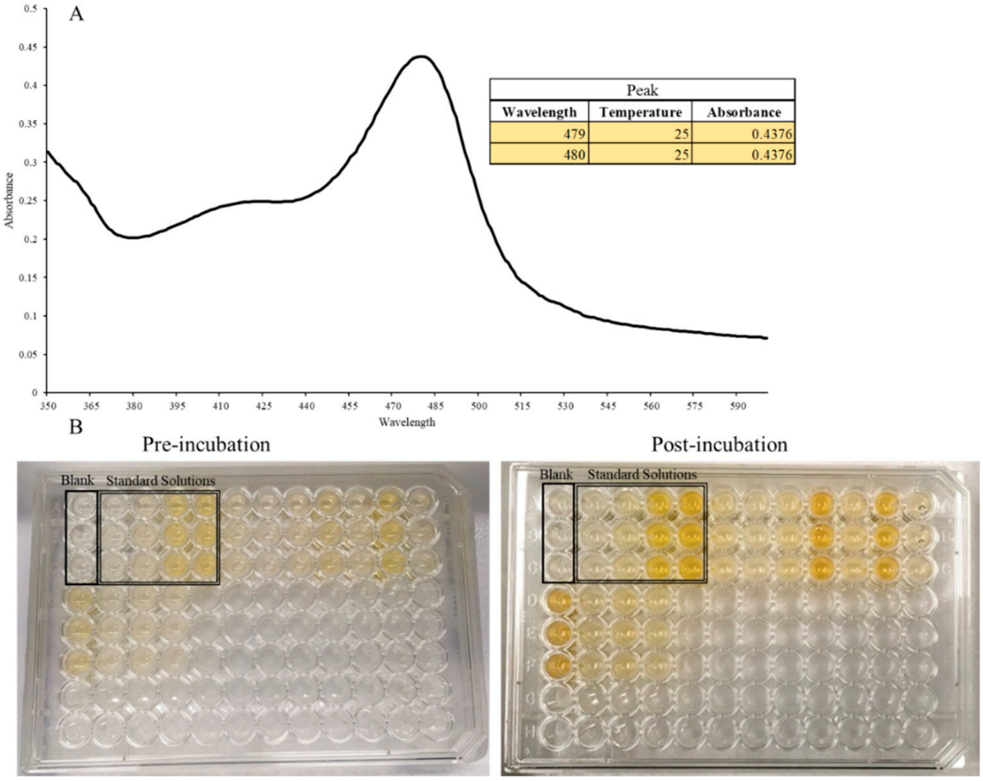

Figure S3. Assay for polysaccharide quantification. (A) Absorbance maximum for the polysaccharide 19F used in the quantification of encapsulation efficiency assays. (B) Colorimetric assay solution development pre- and post-incubation (blank and standard solutions are shown in triplicate and additional wells represent various samples tested within this particular assay).

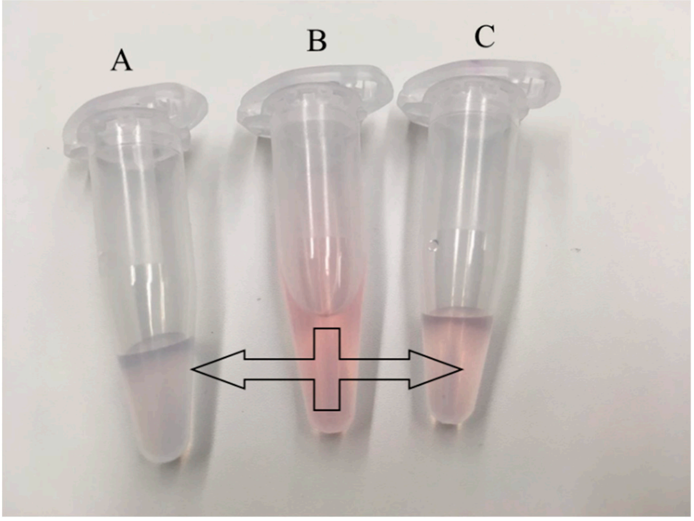

Figure S4. Purification process for the NTA-cobalt liposomal variant featuring purified liposomes (A), liposomes post-extrusion (B), and free polysaccharide (C). Sample analysis was performed using the assay for polysaccharide assessment.
